# Utilizing the VISIA Camera for Analyzing 5-Fluorouracil Treatment Efficacy for Actinic Keratosis

**DOI:** 10.2196/66553

**Published:** 2025-12-19

**Authors:** Emily Woolhiser, Leena Jamal, Jessica Kirk, Bertha Baum, Robert Dellavalle

**Affiliations:** 1 College of Osteopathic Medicine, Kansas City University, 1750 Independence Avenue, Kansas City, MO, 64106, United States, 1 9548494466; 2 College of Osteopathic Medicine, Michigan State University, East Lansing, MI, United States; 3 College of Osteopathic Medicine, Rocky Vista University, Parker, CO, United States; 4 Aventura Dermatology, Aventura, FL, United States; 5 Department of Dermatology, University of Minnesota, Minneapolis, MN, United States

**Keywords:** VISIA camera, medical device, medical equipment, actinic keratosis, 5-fluorouracil, dermatologist, dermatology, skin condition, skin, keratin, case report, keratosis, treatment efficacy

## Abstract

The VISIA camera is a device that captures images of the skin, offering a detailed look at skin health by detecting changes that are often missed during physical exams and by the naked eye. It can help identify changes in UV damage, pigmentation, texture, fine lines, and redness. In dermatology, it has become a useful tool to build targeted treatment plans and follow patient progress over time. We present a case of a male patient diagnosed with diffuse scalp actinic keratoses who was treated with topical 5-fluorouracil (5-FU). VISIA images were taken before treatment, at one week, and again three months following therapy. The images were reviewed for changes in UV spots, texture, and other generalized spots. Results revealed a decrease in UV spots, a temporary improvement in texture followed by a later rise, and no significant change in generalized spots. This case highlights the value of VISIA imaging as an objective method for assessing treatment response and evaluating the effectiveness of 5-FU in the management of actinic keratoses.

## Introduction

The VISIA camera (Canfield) is a novel device that captures images of the skin using a rotating camera with both UV and polarized light to assess the condition of the skin by imaging the surface and subsurface layers. VISIA provides patients with an easy-to-understand analysis, helping them recognize their skin’s problem areas to target further treatment. Skin filters can give patients targeted data on spots, wrinkles, texture, pores, freckles, discoloration, inflammation, and porphyrins. This device is commonly used in cosmetic-oriented settings to tailor skin treatments and build skin care routines [1].

Actinic keratoses (AKs) are precancerous lesions associated with sun exposure that present as rough, scaly patches. AKs are a leading reason for dermatology visits, with a prevalence of 37.5% in White males over 50 years of age [2]. AKs are clinically identified with the naked eye, touch, and the dermatoscope. Cryotherapy with liquid nitrogen is used for single lesions, while field therapy with a strong topical agent can be used for areas with a multitude of lesions [3]. 5-Fluorouracil (5-FU) is a chemotherapeutic agent that is formalized into a cream to treat skin neoplasms and has been shown to be the most efficacious in a trial comparing the four leading field-directed treatments for AKs [4]. However, topical application can cause irritation in the form of inflammation, erythema, crusting, and ulceration, leading to patient reluctance [5]. We believe it would be beneficial to use the VISIA camera for AK patients to get an in-depth look at the effects of sun exposure on their skin. This information could serve as a powerful motivator for individuals who are hesitant to initiate field treatment with 5-FU and may help increase compliance. VISIA could function as a preventive tool by objectively quantifying chronic UV-induced skin damage, thereby reinforcing the importance of therapy and supporting adherence.

## Ethical Considerations

This case report did not require approval from an institutional review board as it is based on patient observations without experimental intervention, in accordance with institutional and local policies. Written informed consent was obtained from the patient for their photographs and medical information to be published. All data were anonymized to protect the patient’s privacy and confidentiality. No compensation was provided to the patient.

## Case Report

In an observational study, a male patient classified as Fitzpatrick skin type II diagnosed with diffuse scalp AKs consented to undergo 5-FU treatment on the scalp and involvement in our study. An initial VISIA image was taken prior to treatment, 5-FU was applied twice daily to the scalp for 4 weeks, and a follow-up photo was obtained 1 week and 3 months after treatment to evaluate lasting efficacy. We used the UV spots filter to assess spots resulting from sun damage (AKs), the spots (non–UV-induced) filter for our control, and the texture filter to analyze the inflammatory process on the skin.

Although our patient had been recommended this therapy previously, he was hesitant to start it due to fear of an exacerbated reaction. He was properly instructed on how to apply the 5-FU cream both morning and night. He had a typical reaction to the 5-FU with erythema, crusting of the lesions, and a mild headache on some days.

**Figure 1.**
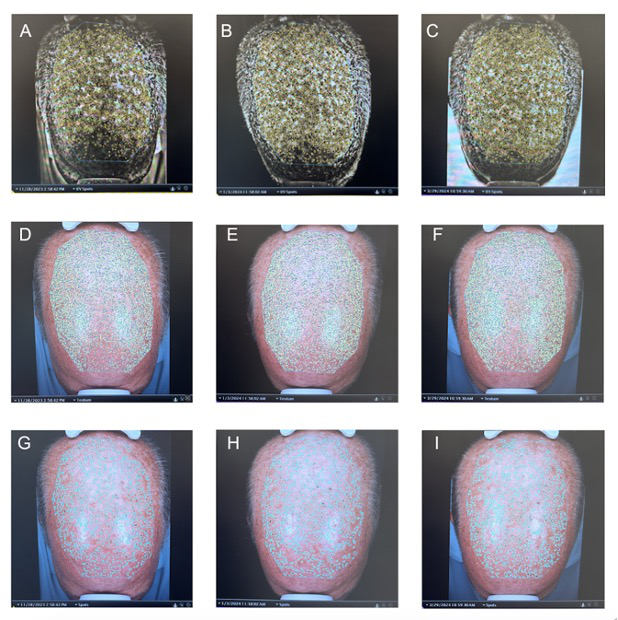
Pretreatment (left column, photos A, D, G), 1 week posttreatment (middle column, photos B, E, H), and 3 months posttreatment (right column, photos C, F, I). Photos with the UV spot filter (top row, photos A, B,C), texture filter (middle row, photos D, E, F), and spot filter (bottom row, photos G, H, I).

Results depicted by the VISIA can be seen in Figure 1 and Table 1. The UV spot filter, which automatically shows the amount of UV sun damage through a multispectral imaging process, showed a feature count decrease from 706 to 676 and percentile reduction from 83% to 42% from day 1 to 1 week posttreatment. For UV spots, a lower percentile is a positive outcome, translating to less sun damage than a larger percentage of people. The spot filter, which acted as our control to affirm the VISIA’s efficacy in identifying UV-damaged areas, produced the same percentile of 9% for all 3 photos at each intervention time. Additionally, we used the texture filter to assess the inflammatory effects of 5-FU. The absolute count showed an initial decrease in texture by both a decreased absolute count and an increased percentile (indicating smoother skin compared to others) 1 week after treatment. The photo taken 3 months after treatment, however, showed an increase in the absolute count but a decrease in the percentile, indicating a rougher texture that could be attributed to the healing process.

**Table 1.** Quantitative analysis as reported by the VISIA. The values in parentheses represent percentile rankings relative to the VISIA reference database.

	UV spots, n (percentile)	Spots, n (percentile)	Texture absolute count, n (percentile)
Pretreatment	706 (83)	563 (9)	9834 (26)
1 week posttreatment	676 (42)	541 (9)	9076 (40)
3 months posttreatment	631 (39)	570 (9)	10,171 (31)

## Discussion

Overall, results favorably depicted the efficacy of 5-FU in treating AKs. Although this treatment has long been regarded as efficacious, this data provided the patient with photographic and numerical evidence of their own skin. To our knowledge, there have not been any other reports of the VISIA camera being used for the purpose of assessing the effects of 5-FU on AKs. An independent investigation assessed the precision of the VISIA on 8 patients for the purpose of imaging in plastic surgery; VISIA precision was satisfactory and patients benefited from the objective data gathered of the skin surface characteristics beyond the subjective assessment [6].

The VISIA has been used to compare products to industry standards and analyze the longevity of products, allowing for objective data to be gathered [7,8]. Another study analyzed melanin and hemoglobin (used as diagnostic markers of skin conditions) by aid of the VISIA as compared to commercial clinical equipment and found high correlation [9]. However, the VISIA’s use remains most concentrated in cosmetics and esthetics, indicating a higher need for trials focused on its medical dermatology use.

The integration of the VISIA camera into general dermatology practice has significant promise for increasing patient compliance and willingness to undergo treatments. Our results demonstrated objective visualization of the UV-induced damage corresponding to AKs and the reduction in damage following treatment. A recent qualitative study revealed that of patients analyzed, half felt that 5-FU treated the AKs and the other half were either unsure or did not think it had any effect. Patients were discouraged by the physical and psychosocial burden, which led to premature discontinuation and refusal for retreatment in future cases [10].

Our patient, who was hesitant of treatment, was encouraged by results seen on his scalp and is open to future treatment. Our study was limited due to its single patient observation and would be strengthened by a longitudinal study with multiple patients. Of note, the percentiles reported are based on an average of patients in the VISIA system corresponding to the preset face mask, not percentiles taken from scalps.

## Conclusion

Continued integration of the VISIA camera into general clinical practice can benefit patients and physicians to serve as an effective tool for creating treatment plans and providing evidence for treatment necessity. While the VISIA camera is a valuable resource, its high cost may limit accessibility in some clinical settings. Nonetheless, we hope this case encourages patient education and empowers patients to better understand the efficacy of 5-FU in treating actinic keratoses.

## Abbreviations

5-FU: 5-fluorouracil
AK: actinic keratosis
